# Supplementary material for: Coastal fish assemblages and predation pressure in northern-central Chilean Lessonia trabeculata kelp forests and barren grounds
Source: PeerJ. 2019 Jun 12;7:e6964. doi: 10.7717/peerj.6964 (PMC6571002; doi:10.7717/peerj.6964)
Supplement: Supplemental Information 1 [file peerj-07-6964-s001.docx]

| Location | Latitude | Longitude | Exploitation regime | Date | Community |
| --- | --- | --- | --- | --- | --- |
| CA - Caleta Angosta | 28° 15’ 46.27’’ S | 71° 10’ 21.37’’ W | MA | 28/02 and 01/03/2017 | KF |
|  |  |  |  | 01-02/03/2017 | KF |
| PC - Pta. Choros | 29° 12’ 10.51’’ S | 71° 29’ 32.52’’ W | MA | 20-21/06/2017 | BG |
|  |  |  |  | 21-22/06/2017 | BG |
|  | 29° 12’ 04.11’’ S | 71° 29’ 23.32’’ W | MA | 15-16/10/2017 | KF |
|  |  |  |  | 16-17/10/2017 | KF |
| CH - Chungungo | 29° 27’ 23.72’’ S | 71° 18’ 32.21’’ W | MA | 8-9/12/2016 | BG |
|  |  |  |  | 19-20/01/2017 | BG |
|  | 29° 26’ 04.46’’ S | 71° 18’ 10.34’’ W | MA | 23-24/11/2016 | KF |
|  |  |  |  | 28-29/12/2016 | KF |
| GU - Guayacancito | 29° 59’ 08.37’’ S | 71° 22’ 41.51’’ W | OA | 29-30/09/2016 | BG |
|  |  |  |  | 27-28/10/2016 | BG |
