# Supplementary material for: Coastal fish assemblages and predation pressure in northern-central Chilean Lessonia trabeculata kelp forests and barren grounds
Source: PeerJ. 2019 Jun 12;7:e6964. doi: 10.7717/peerj.6964 (PMC6571002; doi:10.7717/peerj.6964)
Supplement: Supplemental Information 2 [file peerj-07-6964-s002.docx]

| Site | Date | Community | Prey item | Structure | | Camera | |
| --- | --- | --- | --- | --- | --- | --- | --- |
|  |  |  |  | Vertical | Horizontal | Vertical | Horizontal |
| CA | 28/02 y 01/03/2017 | KF | *T. niger* | 3 | 2 | 3 | 2 |
|  |  |  | *P. laevigatus* | 2 | 3 | 2 | 3 |
|  | 01-02/03/2017 | KF | *T. niger* | 2 | 3 | 1 | 0 |
|  |  |  | *P. laevigatus* | 3 | 2 | 1 | 0 |
| PC | 20-21/06/2017 | BG | *T. niger* | 3 | 2 | 2 | 2 |
|  |  |  | *P. laevigatus* | 2 | 3 | 2 | 2 |
|  | 21-22/06/2017 | BG | *T. niger* | 2 | 3 | 0 | 3 |
|  |  |  | *P. laevigatus* | 3 | 2 | 2 | 1 |
|  | 15-16/10/2017 | KF | *T. niger* | 2 | 2 | 2 | 2 |
|  |  |  | *P. laevigatus* | 3 | 3 | 3 | 2 |
|  | 16-17/10/2017 | KF | *T. niger* | 3 | 3 | 3 | 3 |
|  |  |  | *P. laevigatus* | 2 | 2 | 1 | 2 |
| CH | 8-9/12/2016 | BG | *T. niger* | 3 | 2 | 2 | 1 |
|  |  |  | *P. laevigatus* | 2 | 3 | 2 | 2 |
|  | 19-20/01/2017 | BG | *T. niger* | 2 | 3 | 1 | 2 |
|  |  |  | *P. laevigatus* | 3 | 2 | 3 | 1 |
|  | 23-24/11/2016 | KF | *T. niger* | 3 | 2 | 0 | 2 |
|  |  |  | *P. laevigatus* | 2 | 3 | 1 | 1 |
|  | 28-29/12/2016 | KF | *T. niger* | 2 | 3 | 1 | 2 |
|  |  |  | *P. laevigatus* | 3 | 2 | 3 | 1 |
| GU | 29-30/09/2016 | BG | *T. niger* | 1 | 2 | 1 | 2 |
|  |  |  | *P. laevigatus* | 3 | 3 | 2 | 1 |
|  | 27-28/10/2016 | BG | *T. niger* | 3 | 3 | 1 | 3 |
|  |  |  | *P. laevigatus* | 2 | 2 | 2 | 0 |
