# Supplementary material for: Coastal fish assemblages and predation pressure in northern-central Chilean Lessonia trabeculata kelp forests and barren grounds
Source: PeerJ. 2019 Jun 12;7:e6964. doi: 10.7717/peerj.6964 (PMC6571002; doi:10.7717/peerj.6964)
Supplement: Supplemental Information 3 — Error distributions: BB = beta-binomial, NB = negative binomial, TP = truncated poisson. [file peerj-07-6964-s003.docx]

| Response | Best Model | Zero-inflation | Error | df residual | AICc |
| --- | --- | --- | --- | --- | --- |
| FO vertical | y~habitat+prey+(1\|site)+(1\|fish)+(1\|replicate) | habitat+prey | BB | 359 | 680.26 |
| FO horizontal | y~habitat+prey+(1\|site)+(1\|fish)+(1\|replicate) | habitat+prey | BB | 341 | 867.19 |
| MaxN vertical | count~habitat+prey+(1\|site)+(1\|fish)+(1\|replicate) | habitat | NB | 360 | 394.36 |
| MaxN horizontal | count~habitat+prey+(1\|site)+(1\|fish)+(1\|replicate) | habitat | TP | 343 | 680.44 |
