# Supplementary material for: Coastal fish assemblages and predation pressure in northern-central Chilean Lessonia trabeculata kelp forests and barren grounds
Source: PeerJ. 2019 Jun 12;7:e6964. doi: 10.7717/peerj.6964 (PMC6571002; doi:10.7717/peerj.6964)
Supplement: Supplemental Information 4 — Asterisks show significant effects. SE = standard error. [file peerj-07-6964-s004.docx]

| SR Vertical | | | | |
| --- | --- | --- | --- | --- |
| Fixed effects | Coefficients |  | | |
|  | Estimate | SE | z value | p (>\|z\|) |
| (Intercept) | 0.89364 | 0.21063 | 4.243 | < 0.0001* |
| CA | 0.05348 | 0.42243 | 0.127 | 0.899 |
| GU | -0.8486 | 0.33321 | 0.068 | 0.946 |
| PC | -0.33898 | 0.31033 | -1.124 | 0.261 |
| Kelp Forest | -0.49513 | 0.31033 | -1.595 | 0.111 |
| SR Horizontal | | | | |
| Fixed effects | Coefficients |  | | |
|  | Estimate | SE | z value | p (>\|z\|) |
| (Intercept) | 0.86001 | 0.22669 | 3.794 | < 0.0001* |
| CA | 0.4399 | 0.36192 | 1.215 | 0.224 |
| GU | -0.08682 | 0.35821 | -0.242 | 0.808 |
| PC | 0.11333 | 0.25261 | 0.449 | 0.6536 |
| Kelp Forest | -0.49513 | 0.31033 | -1.096 | 0.272 |
