# Supplementary material for: Coastal fish assemblages and predation pressure in northern-central Chilean Lessonia trabeculata kelp forests and barren grounds
Source: PeerJ. 2019 Jun 12;7:e6964. doi: 10.7717/peerj.6964 (PMC6571002; doi:10.7717/peerj.6964)
Supplement: Supplemental Information 5 — Asterisks show significant effects. SE = standard error. [file peerj-07-6964-s005.docx]

| FO vertical | | | | |
| --- | --- | --- | --- | --- |
| Random effects |  |  |  |  |
| Groups | Name | Variance |  |  |
| Site | intercept | 0.00595 |  |  |
| Fish species | intercept | 10.303 |  |  |
| Replicate | intercept | 0.0214 |  |  |
|  | | | | |
| Fixed effects | Conditional model |  | | |
|  | Estimate | SE | z value | p (>\|z\|) |
| (Intercept) | -7.145 | 1.431 | -4.994 | < 0.0001 * |
| Barren Grounds | 1.044 | 0.226 | 4.625 | < 0.0001 * |
| *Tetrapygus niger* | 0.102 | 0.207 | 0.496 | 0.62 |
|  | Zero-inflation model |  | | |
|  | Estimate | SE | z value | p (>\|z\|) |
| (Intercept) | -1.164 | 0.708 | -1.645 | 0.1 |
| Barren Grounds | -1.611 | 1.2 | -1.342 | 0.18 |
| *Tetrapygus niger* | -1.125 | 1.214 | -0.927 | 0.354 |
