# Supplementary material for: Coastal fish assemblages and predation pressure in northern-central Chilean Lessonia trabeculata kelp forests and barren grounds
Source: PeerJ. 2019 Jun 12;7:e6964. doi: 10.7717/peerj.6964 (PMC6571002; doi:10.7717/peerj.6964)
Supplement: Supplemental Information 6 — Asterisks show significant effects. SE = standard error. [file peerj-07-6964-s006.docx]

| FO horizontal | | | | |
| --- | --- | --- | --- | --- |
| Random effects |  |  |  |  |
| Groups | Name | Variance |  |  |
| Site | intercept | 0.315 |  |  |
| Fish species | intercept | 3.35 |  |  |
| Replicate | intercept | 0.561 |  |  |
|  | | | | |
| Fixed effects | Conditional model |  | | |
|  | Estimate | SE | z value | p (>\|z\|) |
| (Intercept) | -5.295 | 0.761 | -6.956 | < 0.0001 * |
| Barren Grounds | 0.57 | 0.245 | 2.311 | 0.0208 * |
| *Tetrapygus niger* | 0.12 | 0.209 | 0.574 | 0.566 |
|  | Zero-inflation model |  | | |
|  | Estimate | SE | z value | p (>\|z\|) |
| (Intercept) | -2.2 | 1.037 | -2.122 | 0.0338 * |
| Barren Grounds | -16.528 | 4838.588 | -0.003 | 0.997 |
| *Tetrapygus niger* | -17.672 | 8026.687 | -0.002 | 0.998 |
