# Supplementary material for: Coastal fish assemblages and predation pressure in northern-central Chilean Lessonia trabeculata kelp forests and barren grounds
Source: PeerJ. 2019 Jun 12;7:e6964. doi: 10.7717/peerj.6964 (PMC6571002; doi:10.7717/peerj.6964)
Supplement: Supplemental Information 7 — Asterisks show significant effects. SE = standard error. [file peerj-07-6964-s007.docx]

| MaxN vertical | | | | |
| --- | --- | --- | --- | --- |
| Random effects |  |  |  |  |
| Groups | Name | Variance |  |  |
| Site | intercept | < 0.0001 |  |  |
| Fish species | intercept | 7.19 |  |  |
| Replicate | intercept | < 0.0001 |  |  |
|  | | | | |
| Fixed effects | Conditional model |  | | |
|  | Estimate | SE | z value | p (>\|z\|) |
| (Intercept) | -3.819 | 1.176 | -3.248 | 0.0012 * |
| Barren Grounds | 0.972 | 0.203 | 4.793 | < 0.0001 * |
| *Tetrapygus niger* | 0.158 | 0.194 | 0.816 | 0.414 |
|  | Zero-inflation model |  | | |
|  | Estimate | SE | z value | p (>\|z\|) |
| (Intercept) | -26.26 | 188999 | 0 | 1 |
| Barren Grounds | 22.34 | 188999 | 0 | 1 |
| *Tetrapygus niger* | -17.672 | 8026.687 | -0.002 | 0.998 |
