# Supplementary material for: Coastal fish assemblages and predation pressure in northern-central Chilean Lessonia trabeculata kelp forests and barren grounds
Source: PeerJ. 2019 Jun 12;7:e6964. doi: 10.7717/peerj.6964 (PMC6571002; doi:10.7717/peerj.6964)
Supplement: Supplemental Information 8 — Asterisks show significant effects. SE = standard error. [file peerj-07-6964-s008.docx]

| MaxN horizontal | | | | |
| --- | --- | --- | --- | --- |
| Random effects |  |  |  |  |
| Groups | Name | Variance |  |  |
| Site | intercept | < 0.0001 |  |  |
| Fish species | intercept | 2.265 |  |  |
| Replicate | intercept | 0.653 |  |  |
|  | | | | |
| Fixed effects | Conditional model |  | | |
|  | Estimate | SE | z value | p (>\|z\|) |
| (Intercept) | -1.506 | 0.879 | -1.713 | 0.0867 |
| Barren Grounds | 0.63 | 0.31 | 2.032 | 0.0422 * |
| *Tetrapygus niger* | -0.176 | 0.297 | -0.593 | 0.553 |
|  | Zero-inflation model |  | | |
|  | Estimate | SE | z value | p (>\|z\|) |
| (Intercept) | 1.122 | 0.178 | 6.316 | < 0.0001* |
| Barren Grounds | -0.194 | 0.243 | -0.8 | 0.424 |
| *Tetrapygus niger* | -17.672 | 8026.687 | -0.002 | 0.998 |
