# Supplementary material for: Coastal fish assemblages and predation pressure in northern-central Chilean Lessonia trabeculata kelp forests and barren grounds
Source: PeerJ. 2019 Jun 12;7:e6964. doi: 10.7717/peerj.6964 (PMC6571002; doi:10.7717/peerj.6964)
Supplement: Supplemental Information 9 — Asterisks show significant effects. SE = standard error. [file peerj-07-6964-s009.docx]

| Fixed effects | Coefficients |  | | |
| --- | --- | --- | --- | --- |
|  | Estimate | SE | z value | p (>\|z\|) |
| (Intercept) | 1.1408 | 0.1286 | 8.869 | < 0.0001* |
| Kelp forest | -0.1929 | 0.1281 | -1.506 | 0.132 |
| *Tetrapygus niger* | -0.8486 | 0.1327 | -6.396 | < 0.0001* |
| Replicate | intercept | 0.653 |  |  |
