# Supplementary material for: Coastal fish assemblages and predation pressure in northern-central Chilean Lessonia trabeculata kelp forests and barren grounds
Source: PeerJ. 2019 Jun 12;7:e6964. doi: 10.7717/peerj.6964 (PMC6571002; doi:10.7717/peerj.6964)
Supplement: Supplemental Information 10 [file peerj-07-6964-s010.docx]

| Species | Kelp forest | | Barren grounds | |
| --- | --- | --- | --- | --- |
|  | *T. niger* | *P. laevigatus* | *T. niger* | *P. laevigatus* |
| *Scartichthys viridis/gigas* | 0 | 7 | 0 | 25 |
| *Cheilodactylus variegatus* | 0 | 7 | 0 | 0 |
| *Homalaspis plana* (crab) | 0 | 0 | 1 | 0 |
| *Romaleon setosum* (crab) | 0 | 0 | 1 | 0 |
