# Supplementary figures and images for: Coastal fish assemblages and predation pressure in northern-central Chilean Lessonia trabeculata kelp forests and barren grounds
Source: PeerJ. 2019 Jun 12;7:e6964. doi: 10.7717/peerj.6964 (PMC6571002; doi:10.7717/peerj.6964)

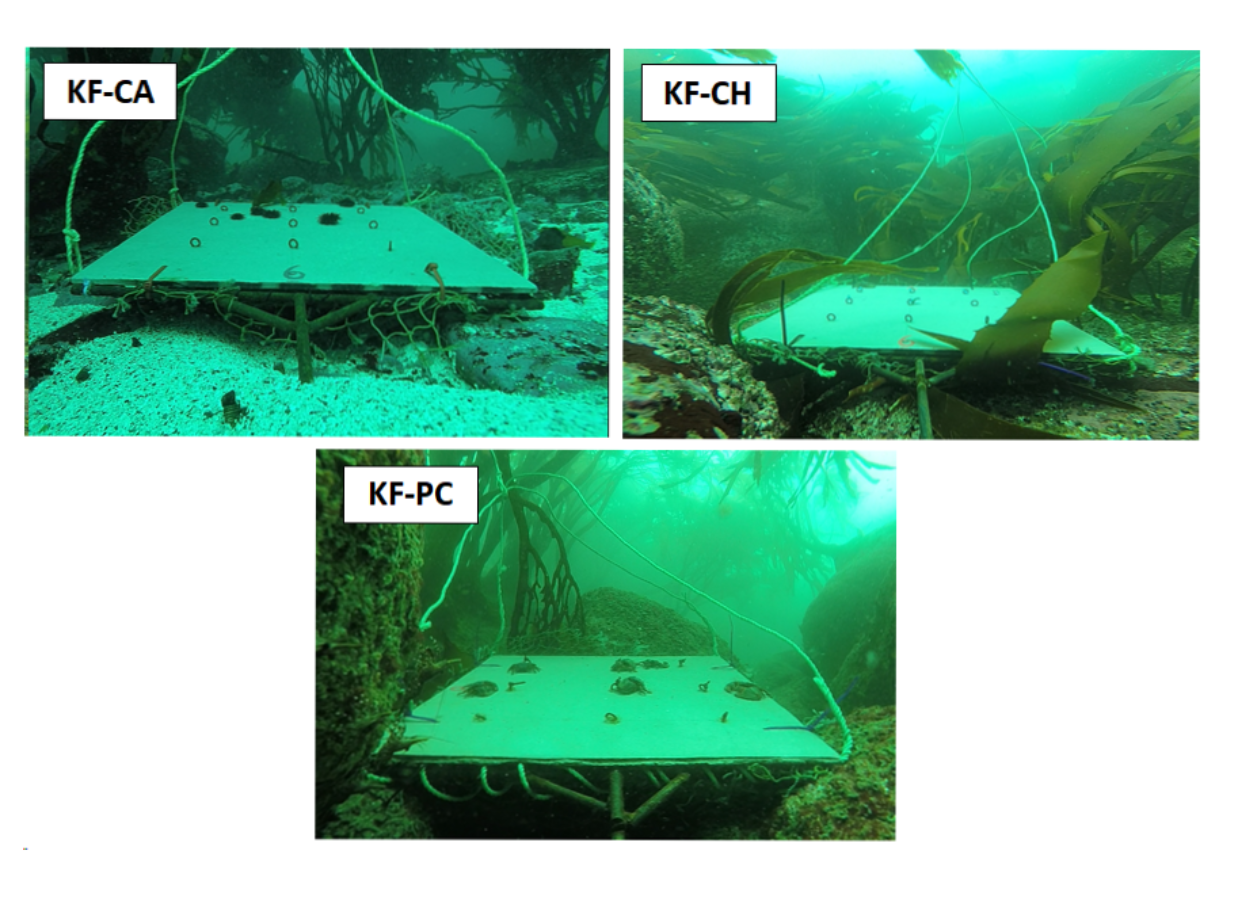

Supplement: Supplemental Information 11 — Images show zones where the structures were usually deployed allowing to view the typical densities of the kelps. Photo Credits: Nicolás Riquelme-Pérez. [file peerj-07-6964-s011.png]

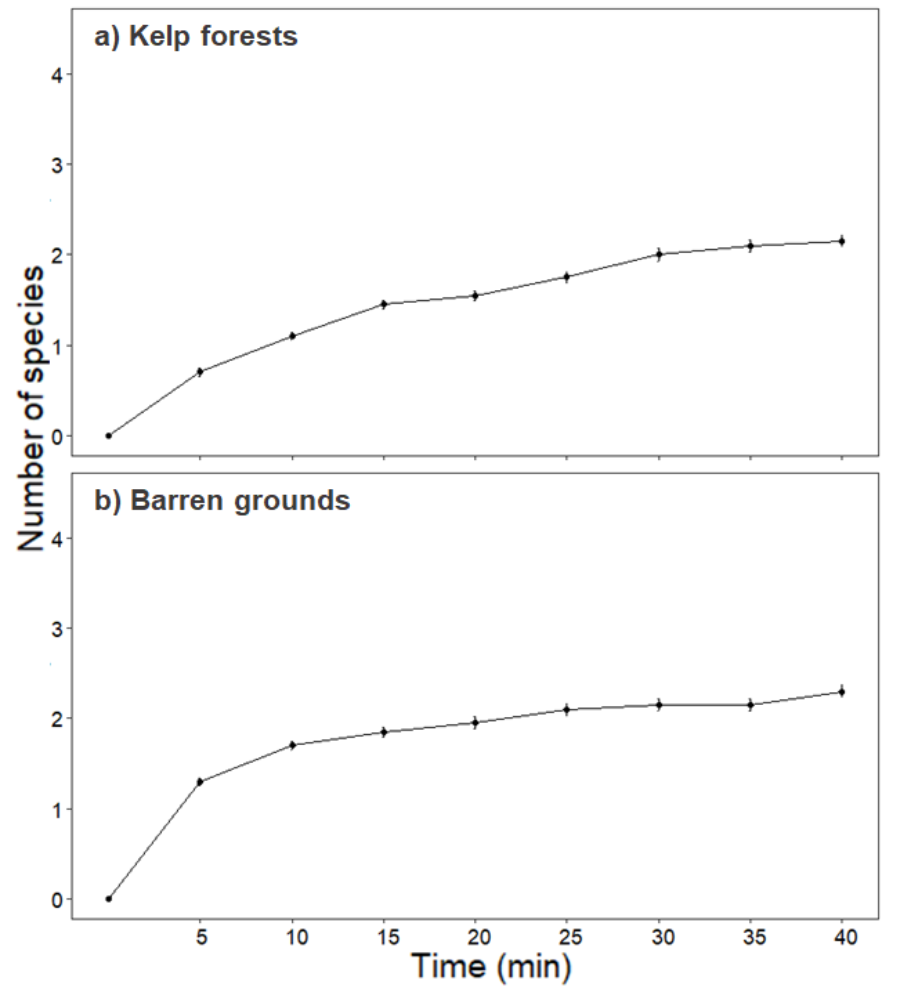

Supplement: Supplemental Information 12 — The different study sites (KF-CA, KF-PC, KF-CH; BG-PC, BG-CH, BG-GU) were pooled for this figure. [file peerj-07-6964-s012.png]
